# Intradialytic Hypotension in Critically Ill Patients on Hemodialysis With A-Line versus B-Line Pattern on Lung Ultrasonography

**DOI:** 10.1016/j.ekir.2021.04.010

**Published:** 2021-04-26

**Authors:** Yuriy Khanin, Jamie S. Hirsch, Daniel Stalbow, Meng Zhang, Zubair Hasan, Daniel W. Ross

**Affiliations:** 1 Division of Kidney Diseases and Hypertension, Donald and Barbara Zucker School of Medicine at Hofstra/Northwell, Hempstead, New York, USA; 2 Feinstein Institutes for Medical Research, Donald and Barbara Zucker School of Medicine at Hofstra/Northwell, Hempstead, New York, USA; 3 Division of Pulmonary, Critical Care, and Sleep Medicine, Donald and Barbara Zucker School of Medicine at Hofstra/Northwell, Hempstead, New York, USA

**Keywords:** POCUS, lung ultrasound, critical care, A-lines, B-lines, intradialytic hypotension

Adequate volume removal in critically ill patients during hemodialysis can be limited due to intradialytic hypotension (IDH). Point of care ultrasonography (POCUS) is a reliable tool to aid in volume assessment. When an ultrasound wave passes the pleura into a normally aerated lung, the high acoustic mismatch causes some of the waves to reverberate. This creates a horizontal reverberation artifact, that when seen in all examined rib interspaces, is termed an “A-line pattern.” If an ultrasound wave meets a water-thickened interlobular septa beneath the pleural surface, the waves are reverberated in an air-fluid microenvironment, creating a linear reverberation artifact known as a “B-line.” A “B-line pattern” is noted if 3 or more B-lines are seen at 2 interspaces on both the left and the right hemi-thorax.

A-line pattern on lung ultrasound (LUS) predicts a low pulmonary artery occlusion pressure, whereas the number of B-lines indicate increased extravascular lung water.^1^^,^^2^ Literature on LUS in patients with kidney disease has focused on quantifying the number of B-lines^3^; however, clinicians infrequently count or document the number of B-lines, preferring instead to report LUS findings according to a qualitative assessment suggested in international consensus guidelines.^4^ LUS findings might facilitate more accurate volume assessment and therefore enable nephrologists to refine their ultrafiltration prescription. In this study, we hypothesize that patients with A-line pattern (dry lungs) on LUS have a higher incidence of IDH compared with patients with B-line pattern (wet lungs). Our methods are available in a supplementary file.

We identified 113 intensive care unit (ICU) patients with LUS findings of either A-line pattern or B-line pattern on the same day as an hemodialysis procedure. Patients with lung pathologies such as acute respiratory distress syndrome or pneumonia were excluded from this study. Patients with B-line pattern were likely to have cardiogenic pulmonary edema. The mean age was 63 and the population was 66% male; 43% of patients were septic and 52% required mechanical ventilation. Details about the patient population as well as our univariable and multivariable analysis are displayed in Table 1. On univariable analysis, patients with A-line pattern on LUS were more likely to experience IDH compared with patients with B-line pattern (odds ratio [OR]: 3.63; 95% confidence interval [CI]: 1.40–9.40). Patients not receiving invasive mechanical ventilation were less likely to experience IDH (OR: 0.41; 95% CI: 0.19–0.88). Acute Physiologic Assessment and Chronic Health Evaluation (APACHE) score was associated with a higher odds of experiencing IDH (OR: 1.02; 95% CI: 1.01–1.04). In a multivariable model, both APACHE score and LUS findings were significantly associated with IDH; however, the magnitude of the effect on IDH was greater for LUS than for APACHE score.

**Table 1:** Baseline characteristics of study cohort, by lung ultrasound findings

Variable	Total (*N* = 113) Mean (SD) or Frequency (%)	A-line (*n* = 84) Mean (SD) or Frequency (%)	B-line (*n* = 29) Mean (SD) or Frequency (%)	*P* value
Age	63.14 (16.45)	63.24 (16.29)	62.86 (17.35)	0.99
Sex				
Female	47 (41.59%)	34 (40.48%)	13 (44.83%)	0.68
Male	66 (58.41%)	50 (59.52%)	16 (55.17%)	
Race				
White	30 (26.55%)	23 (27.38%)	7 (24.14%)	
African American	46 (40.71%)	35 (41.67%)	11 (37.93%)	0.91
Asian	21 (18.58%)	15 (17.86%)	6 (20.69%)	
Other/Multiracial	14 (12.39%)	10 (11.90%)	4 (13.79%)	
Unknown	2 (1.77%)	1 (1.19%)	1 (3.45%)	
Comorbidities				
ESKD	66 (58.41%)	46 (54.76%)	20 (68.97%)	0.18
HFpEF	19 (16.81%)	15 (17.86%)	4 (13.78%)	0.61
HFrEF	24 (21.24%)	19 (22.62%)	5 (17.24%)	0.54
CAD	45 (39.82%)	34 (40.48%)	11 (37.93%)	0.80
CVA	22 (19.64%)	15 (18.07%)	7 (24.14%)	0.48
DM	57 (50.44%)	43 (51.19%)	14 (48.28%)	0.79
HTN	95 (84.07%)	69 (82.14%)	26 (89.66%)	0.34
Malignancy	24 (21.24%)	15 (17.86%)	9 (31.03%)	0.13
Clinical characteristics				
Sepsis	43 (38.05%)	32 (38.10%)	11 (37.91%)	0.98
Mechanical ventilation	52 (46.02%)	43 (51.19%)	9 (31.03%)	0.06
Systolic blood pressure >100 mm Hg before dialysis	109 (96.46%)	82 (97.62%)	27 (93.10%)	0.26
Dialysis treatment				
Mean blood flow (ml/min)	332.30 (71.94)	329.17 (71.22)	341.38 (74.49)	0.39
Mean dialysate flow (ml/min)	503.54 (59.66)	504.76 (57.88)	500 (65.47)	0.72
Sodium modeling	11 (9.73%)	9 (10.71%)	2 (6.90%)	0.54
Prescribed ultrafiltration (ml)	1514.16 (1109.36)	1330 (1117)	2048 (909)	0.002
Delivered ultrafiltration (ml)	1254.12 (1060.66)	1041 (1026)	1872 (922)	< 0.001
Intradialytic hypotension	52 (46.02%)	45 (53.57%)	7 (24.14%)	0.01
APACHE	87.19 (27.62)	91.23 (28.38)	75.76 (22.00)	0.01

Univariable analysis		
Variable	Odds ratio (95% CI)	*P* value
Age	1.02 (0.99–1.04)	0.22
Sex		
Male (reference)	1	
Female	0.79 (0.31–1.67)	0.53
Race		0.4939
White (reference)	1	
African American	0.47 (0.18–1.20)	
Asian	0.500 (0.16–1.55)	
Other/Multiracial	0.67 (0.04–11.76)	
Unknown	0.37 (0.10–1.38)	
Comorbidities		
ESKD	0.78 (0.37–1.67)	0.53
HFpEF	0.94 (0.35–2.51)	0.90
HFrEF	1.00 (0.41–2.50)	0.98
CAD	0.63 (0.30–1.31)	0.21
CVA	1.26 (0.49–3.25)	0.63
DM	0.58 (0.28–1.23)	0.16
HTN	0.53 (0.18–1.54)	0.24
Malignancy	1.56 (0.62–3.92)	0.35
Clinical characteristics		
Sepsis	0.72 (0.33–1.53)	0.39
Mechanical ventilation	0.41 (0.19–0.88)	0.03
Systolic blood pressure >100 mm Hg before dialysis	0.27 (0.03–2.7)	0.27
Dialysis treatment		
Mean blood flow (ml/min)	1.00 (0.99–1.01)	0.27
Mean dialysate flow (ml/min)	1.00 (0.99–1.00)	0.96
Sodium modeling	0.45 (0.12–1.64)	0.23
Lung ultrasound		
A-line or B-line (A-line vs. B-line)	3.63 (1.40–9.40)	0.01
APACHE	1.02 (1.01–1.04)	0.01

Multivariable analysis			
Variable		Odds ratio (95% CI)	*P* value
A line or B line	A line vs. B line	3.01 (1.10–8.22)	0.03
Age		1.01 (0.98–1.04)	0.46
Gender	Female vs. Male	0.68 (0.29–1.58)	0.36
ESKD	Yes vs. No	1.66 (0.73–3.78)	0.23
APACHE		1.02 (1.00–1.03)	0.04

APACHE, Acute Physiologic Assessment and Chronic Health Evaluation; CAD, coronary artery disease; CI, confidence interval; CVA, cerebrovascular accident; DM, diabetes mellitus; ESKD, end-stage kidney disease; HFpEF, heart failure with preserved ejection fraction; HFrEF, heart failure with reduced ejection fraction; HTN, hypertension.

We found that ICU patients with A-line pattern are significantly more likely to experience IDH compared with patients with B-line pattern (OR: 3.01; 95% CI: 1.10–8.22), even after adjustment for APACHE score. Prior research has shown the utility of LUS in identifying patients at risk of IDH. In a study by de Hora Passos *et al.*^5^ using 28-point LUS on 248 critically ill patients requiring kidney replacement therapy, patients with more than 14 B-lines were characterized as having pulmonary congestion. Almost half (48%) of patients with pulmonary congestion experienced IDH, compared with 67% without pulmonary congestion.^5^ As a comprehensive 28-point LUS study can take between 6 and 15 minutes,^6^ its utility in clinical practice may remain limited. Our findings that patients with A-line pattern are more likely to experience IDH are congruent with the report of de Hora Passos *et al.*,^5^ yet may be more clinically applicable. Indeed, our findings support clinical practice guidelines that recommend 8-point LUS to qualitatively characterize lung pathology, whereas many research studies focus on quantifying the number of B-lines.^3^^,^^4^

Our study has several limitations, including its retrospective design and limited sample size. In our ICUs, the typical workflow is that nephrology rounding happens concurrently with ICU rounding and POCUS examination, with dialysis prescriptions written afterward. The documentation time of POCUS examination in the medical record is variable, and we cannot be certain about the timing of POCUS examination in relationship to dialysis. Given the rounding schedule, we expect that POCUS occurs before dialysis in the vast majority of cases. Given the retrospective nature of this study, we also do not know the extent to which the prescribing nephrologist was aware of the POCUS results. In addition, we excluded patients with other LUS pathology, limiting generalizability of results. A final and important limitation of our study was that we did not incorporate cardiac findings. In practice, many intensivists incorporate echo findings into their volume assessment; however, the purpose of this study was to see if a qualitative approach to LUS can predict IDH. Our results suggest that it can. Regardless, future prospective studies should include echo findings as well.

In conclusion, we found that among ICU patients receiving dialysis, those with A-line pattern were more likely to experience IDH than those with B-line pattern on POCUS. Our data build on other research,^5^ showing a strong signal that LUS findings can predict IDH, and can potentially be used to better personalize dialysis ultrafiltration prescription. These findings need to be corroborated by additional prospective studies to examine the impact on clinical practice.

## Disclosures

JSH reports personal fees from Boehringer Ingelheim, outside the submitted work. All the other authors declared no competing interests.
