# Supplementary material for: Intradialytic Hypotension in Critically Ill Patients on Hemodialysis With A-Line versus B-Line Pattern on Lung Ultrasonography
Source: Kidney Int Rep. 2021 Apr 26;6(7):1969–72. doi: 10.1016/j.ekir.2021.04.010 (PMC8258452; doi:10.1016/j.ekir.2021.04.010)
Supplement: Supplementary File (PDF) [file mmc1.pdf]

We conducted a retrospective observational cohort study from two tertiary hospitals in a New York health system. Data were obtained from the medical intensive care units (ICU) using the enterprise inpatient electronic health record (EHR), Sunrise Clinical Manager (Allscripts, Chicago, IL). All adult (age ≥18) patients with an EHR documentation for point of care ultrasound (POCUS) between January 1, 2016, and September 23, 2019, and who underwent a hemodialysis (HD) procedure on the same day were eligible.

We identified 259 instances fulfilling the criteria. For patients who had multiple POCUS procedures and HD treatments, we included the first HD treatment (94 instances excluded), leaving 165 unique patients. Following exclusion for POCUS without lung ultrasound (LUS) [21 patients], presence of interstitial lung disease including ARDS or pneumonia diagnosed by POCUS or other imaging (11), and patients where LUS findings were not clearly stated as A-line or B-line pattern (20), 113 patients remained for analysis. The Institutional Review Board of Northwell Health approved the study protocol.

We manually reviewed patient charts and collected data on demographics, comorbid conditions, invasive mechanical ventilation, vasopressor dose, vital signs, and details of the HD procedure. APACHE score from the time of admission to the ICU was also collected. The outcome of interest was intradialytic hypotension (IDH), defined as a decrease in systolic blood pressure ≥20 mmHg with any of:

1. A failure to meet prescribed ultrafiltration (UF) goal;
2. Initiation of vasopressors or an increase in the dose requirement during HD treatment;
3. Development of symptoms consistent with IDH (such as abdominal discomfort, nausea, vomiting, muscle cramps, dizziness or fainting).

During morning ICU rounds, a house officer performed whole body ultrasound. Ultrasounds were either performed by house staff with formal training or who were on an ultrasound elective. Regardless, all ultrasounds were performed under the direct supervision of an attending physician. These attendings either teach critical care ultrasonography, including lung ultrasound, at the American College of Chest Physicians, or are certified in Critical Care Echocardiography by the National Board of Echocardiography. A variable number but at least eight rib interspaces were scanned with careful attention to the pleural line. For analysis, we included patients with either A-line or B-line pattern. In particular patients with irregular pleural lines were excluded. While cardiac images were obtained and documented only lung ultrasound findings were analyzed and extracted in this particular study. Following rounds, images were uploaded to a central server and interpreted by the attending intensivist. The intensivist's report of findings were then uploaded to the EHR. Nephrologists' determination of volume status and ultrafiltration prescription were determined based upon clinical evaluation and available objective data including vital sign trends and laboratory values.

Data were summarized using descriptive statistics. Categorical variables were summarized using frequencies and percentages; continuous variables were summarized using mean and standard deviation. Categorical variables were compared between A-line pattern and B-line pattern using Chi-squared or Fisher's exact test; and continuous variables were compared using two-sample t-test or Wilcoxon rank sum test. Logistic regression analyses were performed to assess the association between LUS findings and IDH. Univariable analyses were performed first, followed by multivariable analysis. In the final logistic regression model, A-line or B-line, age, gender, ESKD status, and APACHE score were included. Results were reported as adjusted odds ratios (ORs) with 95% confidence intervals (CIs).
